# Fibrin-based factor delivery for therapeutic angiogenesis: friend or foe?

**DOI:** 10.1007/s00441-022-03598-w

**Published:** 2022-02-17

**Authors:** Ludovic Melly, Andrea Banfi

**Affiliations:** 1 Department of Biomedicine, Basel University Hospital and University of Basel, Basel, Switzerland; 2 Cardiac, Vascular and Thoracic Surgery Department, UCLouvain, CHU UCL Namur, Yvoir, Belgium

**Keywords:** Neovascularization, Vascular endothelial growth factor, Extracellular matrix, Ischemia, Fibrosis

## Abstract

Therapeutic angiogenesis aims at promoting the growth of blood vessels to restore perfusion in ischemic tissues or aid tissue regeneration. Vascular endothelial growth factor (VEGF) is the master regulator of angiogenesis in development, repair, and disease. However, exploiting VEGF for therapeutic purposes has been challenging and needs to take into account some key aspects of VEGF biology. In particular, the spatial localization of angiogenic signals within the extracellular matrix is crucial for physiological assembly and function of new blood vessels. Fibrin is the provisional matrix that is universally deposited immediately after injury and supports the initial steps of tissue regeneration. It provides therefore several ideal features as a substrate to promote therapeutic vascularization, especially through its ability to present growth factors in their physiological matrix-bound state and to modulate their availability for signaling. Here, we provide an overview of fibrin uses as a tissue-engineering scaffold material and as a tunable platform to finely control dose and duration of delivery of recombinant factors in therapeutic angiogenesis. However, in some cases, fibrin has also been associated with undesirable outcomes, namely the promotion of fibrosis and scar formation that actually prevent physiological tissue regeneration. Understanding the mechanisms that tip the balance between the pro- and anti-regenerative functions of fibrin will be the key to fully exploit its therapeutic potential.

## Therapeutic angiogenesis

Ischemia is caused by inadequate balance between oxygen supply and demand. When happening in the heart, patients suffer from angina if the imbalance remains transient, or they undergo a myocardial infarction (MI) in case a complete occlusion occurs. Ischemic heart disease remains still the most common cause of death worldwide (Virani et al. 2021). Although angioplasty and surgery are available to restore the vascular flow distal to a stenosis, therapeutic angiogenesis can help as a concomitant therapy or even emerge as a stand-alone therapy if no other clinical intervention is feasible.

Therapeutic angiogenesis is broadly defined as a treatment strategy that promotes the growth of blood vessels to restore perfusion in ischemic tissues. Depending on where the process takes place a distinction should be made between angiogenesis and arteriogenesis. *Angiogenesis* is defined as the expansion of a tissue micro-vascular network starting from the pre-existing capillaries, which have a diameter of 8–12 µm and are responsible for the metabolic exchanges. The most important physiological inducer of angiogenesis is ischemia. On the other hand, *arteriogenesis* refers to the formation of larger arterial vessels that channel the blood flow around the occlusion site as collaterals. This process instead consists of the enlargement and subsequent maturation of pre-existing small and poorly perfused collateral vessels that is induced mainly by shear stress and inflammatory cells but not directly by hypoxia. The two phenomena are related, since angiogenesis in downstream capillary beds directly stimulates upstream collateral remodeling and arteriogenesis (Annex 2013). The expansion of the micro-vascular bed, induced by angiogenic growth factors, is capable of causing the enlargement of upstream collateral arteries through both increased shear stress and gap junction-mediated retrograde signaling along vessel walls, thereby ensuring that blood perfusion downstream of the occlusion is restored by way of a biological bypass (Pries et al. 2010; Rissanen et al. 2005).

Therapeutic angiogenesis finds application in two distinct fields in regenerative medicine: (i) restoration of blood flow to ischemic tissue and (ii) rapid vascularization of tissue-engineered grafts. In the first case, ischemia derives from a chronic or acute impairment of blood flow to tissue, leading to a loss of function and even necrosis. The most prevalent forms of ischemic disease are the result of progressive atherosclerosis with stenosis of major arteries in the lower limbs (peripheral artery disease or PAD) or in the heart (coronary artery disease or CAD). Diffuse microvascular impairment is an additional mechanism of tissue ischemia in type 2 diabetes, leading to non-healing cutaneous ulcers that are a major cause of morbidity in these patients (Virani et al. 2021). In these conditions, the promotion of vascular growth aims at restoring the blood supply to the chronically ischemic tissue that is still viable but dysfunctional, such as the hibernating myocardium in the border zone of an infarction.

On the other hand, tissue engineering aims at generating tissue replacements by combining suitable populations of progenitor cells with a biomaterial-based scaffold, with or without the addition of bioactive components, such as growth factors (Ahmed et al. 2008). Significant advances in stem cell biology and biomaterial engineering have made it possible to generate replacements of almost any tissue in the body. However, a major limiting factor for the clinical translation of this approach is the need for the rapid vascularization of the grafts upon in vivo implantation, to ensure the survival, differentiation, and function of the seeded progenitors inside the thick tissue-engineered constructs, since their clinically relevant size makes it impossible for oxygen and nutrients to reach deeper than about 1 mm by simple diffusion from surrounding vessels (Gianni-Barrera et al. 2020).

## Fundamental biological concepts in angiogenesis

Vascular endothelial growth factor (VEGF) is the master regulator of angiogenesis, as this family of factors controls both physiological and pathological growth of blood and lymphatic vessels by signaling through their cognate receptors. The mammalian VEGF family comprises five main ligands (VEGF-A, -B, -C, and -D and placenta-derived growth factor, PlGF) and three receptors (VEGF-R1, -R2, and -R3). While the principal role of VEGF-C and -D is to stimulate lymphatic angiogenesis through VEGF-R3, blood vessel growth is mostly coordinated by the signaling of VEGF-A and -B and PlGF through R1 and R2.

VEGF is the most specific single factor capable of initiating the complex cascade of events leading to angiogenesis. Inactivation of VEGF during development results in embryonic lethality (Ferrara et al. 1996; Haigh et al. 2000), whereas VEGF delivery has been widely shown to induce new vascular growth in a variety of therapeutic models (Yla-Herttuala et al. 2017). The best understood mechanism of angiogenesis is sprouting, which requires the formation of VEGF gradients in the microenvironment around each producing cell, thanks to its ability to bind to extracellular matrix (ECM) (Park et al. 1993). The orderly regulation of this morphogenic process requires endothelial cells to specialize into either migrating tip or proliferating stalk cells (Gerhardt et al. 2003) and is controlled by Notch signaling (Hellstrom et al. 2007). The first cells that respond to VEGF in the matrix become tip cells, which do not proliferate and are not involved in the formation of lumen structures, but rather sense the gradient of VEGF in the microenvironment and migrate towards it, thereby initiating the sprouting process. In response to VEGF, tip cells upregulate expression of Delta-like-4 (Dll4), which activates Notch-1 signaling on the neighboring endothelial cells and instructs them to acquire a stalk cell phenotype, instead. Contrary to tip cells, stalk cells respond to the total concentration of VEGF by proliferating rather than migrating and form new lumenized structures behind the sprouting tip.

However, sprouting is not the only process by which angiogenesis takes place. An alternative mechanism of vascular network expansion is intussusception, also referred to as splitting angiogenesis (Gianni-Barrera et al. 2014). Initially considered a kind of anatomical curiosity taking place only during the development of some organs, such as the lung and the kidney, intussusceptive angiogenesis has been increasingly recognized over the last decade as a common mechanism of vascular growth, with important therapeutic implications (Gianni-Barrera et al. 2020). Intussusception can be initiated very rapidly by increased blood flow and shear stress in the absence of growth factors (Egginton et al. 2001). However, very importantly, it is also the principal mechanism by which VEGF delivery at therapeutically relevant doses induces angiogenesis in clinically relevant tissues like skeletal muscle (Gianni-Barrera et al. 2013). How VEGF may induce sprouting or intussusception remains unclear, but its distribution in the matrix appears to be an important factor and the absence of a concentration gradient favors intussusception. This is suggested by comparing different outcomes of VEGF expression at different doses in the same tissue of skeletal muscle. In fact, spontaneous upregulation of VEGF at physiologically limited levels by ischemia leads to sprouting (Al Haj Zen et al. 2010), whereas its over-expression at significantly higher and therapeutic levels, which saturate the scarce matrix between muscle fibers and abrogate local gradients, induces angiogenesis by intussusception (Gianni-Barrera et al. 2013). During intussusceptive angiogenesis, endothelial cells respond to VEGF exclusively by proliferation without migration: no tip cells are formed and proliferating endothelium behaves functionally only as stalk cells. This leads to circumferential enlargement of the vessel, which then splits longitudinally into new vascular structures. Splitting requires the formation of endothelial pillars across the vascular lumen, which derive either from a vascular wall invagination that creates a contact between the opposite endothelial cells (Makanya et al. 2009), or by the extension and fusion of intraluminal filopodial-like protrusion from the endothelium (Egginton 2009). In both cases, subsequently, the endothelial junctions reorganize at the contact points and myofibroblast invade the core, stabilizing the structures into mature transluminal tissue pillars. Finally, these align longitudinally, fuse with each other, and split the affected vascular segment.

At the end of these morphogenic processes, newly formed endothelial structures are unstable and susceptible to rapid regression if VEGF signaling is interrupted. Vascular maturation and stabilization are the processes by which endothelial cells return to quiescence and independence from continued VEGF signaling and require the physical association of endothelium with a population of mesenchymal cells called pericytes. These are recruited by platelet-derived growth factor-BB (PDGF-BB), produced by activated endothelium, establish tight cell-to-cell contact with endothelial cells by residing under their basal lamina, and regulate their function through a complex cross-talk involving both paracrine and cell contact-dependent signaling pathways (Reginato et al. 2011). An active role in vascular maturation is also played by a recently identified population of myeloid cells (Giacca and Zacchigna 2012), which are recruited by Semaphorin-3a signaling through its receptor Neuropilin-1 and are therefore called neuropilin-expressing monocytes (NEM), and produce a host of pro-maturation paracrine factors, including PDGF-BB, Angiopoietin-1, and transforming growth factor-β1 (TGF-β1) (Groppa et al. 2015).

## Therapeutic requirements for angiogenic factor delivery

During ischemia, hypoxic parenchymal cells are the main source of VEGF production, e.g., myofibers in skeletal muscle or cardiomyocytes in the heart (Banfi and Gianni-Barrera 2015; Braile et al. 2020). However, ischemia-induced upregulation of VEGF is tightly limited by the regulatory sequences in the endogenous promoter and this response may sometimes be insufficient to restore flow, necessitating delivery at supra-physiological levels in order to obtain a therapeutic effect. For example, a study of cell-based VEGF over-expression in ischemic muscle showed that functional restoration of blood flow required VEGF levels at least sixfold greater than those achieved by endogenous upregulation alone (von Degenfeld et al. 2006).

The selected aspects of the biology of vascular growth described above carry significant implications for the use of VEGF in therapeutic angiogenesis, particularly with respect to its dose, distribution in tissue, and duration of expression. In fact, it is well-known that uncontrolled over-expression of VEGF can induce severely aberrant vasculature, and even the growth of angioma-like vascular tumors in a variety of tissues, such as skeletal muscle (Ozawa et al. 2004; Springer et al. 1998), subcutaneous fat (Sundberg et al. 2001), the liver (Kitajima et al. 2005; Leppanen et al. 2006), and the heart (Lee et al. 2000; Schwarz et al. 2000). The discovery of VEGF sparked an immediate interest in its therapeutic potential and intense clinical investigation took place in the 1990s and 2000s to treat cardiovascular ischemia in both coronary and peripheral artery disease with VEGF delivery as a protein or by gene therapy vectors. However, after some promising initial reports, controlled clinical trials have generally failed to demonstrate therapeutic efficacy at safe vector doses, and clinical investigation of VEGF in cardiovascular disease has essentially stopped and reverted to preclinical experimentation. This first generation of cardiovascular VEGF clinical trials is discussed in several excellent reviews (Cooke and Losordo 2015; Rubanyi 2013). Later, VEGF protein delivery has also been investigated for the treatment of chronic foot ulcers in diabetic patients, and a product is clinically approved in some countries (telbermin), but efficacy is limited and approval has been revoked by the US FDA (Berry-Kilgour et al. 2021). A major issue limiting VEGF efficacy is that its therapeutic dosing, e.g., by gene delivery, has proven to be very challenging, with lower doses of gene therapy vectors being inefficacious and higher doses rapidly causing aberrant vascular growth (Rubanyi 2013). Subsequent studies revealed that this difficulty is related in part to one of the properties of VEGF that is crucial for its very biological function, namely its affinity for ECM. In fact, since VEGF remains tightly localized in the microenvironment around each producing cell after being secreted, different growth factor concentrations do not average with each other, even across distances of tens or hundredths of micrometers. Consequently, if expression is excessive in even just some areas, this is enough to cause aberrant vascular growth even if the total VEGF dose is rather low. However, if a homogeneous distribution of factor or of expression levels can be achieved, then controlled physiological angiogenesis is possible (Gianni-Barrera et al. 2020). This concept was clearly shown by studies using myoblast populations stably transduced with retroviral vectors to express VEGF in skeletal muscle (Ozawa et al. 2004). After retroviral transduction, different cells express a variety of levels due to differing viral copy numbers and their sites of chromatin integration. When the heterogenous transduced population was implanted, aberrant angioma-like structures were always induced, even if diluting it with non-expressing cells until very low total levels. However, by implanting monoclonal populations, derived from single cells isolated from the same heterogeneous population, in which every cell produced the same amount, it became clear that a wide range of VEGF levels exists that induce only normal, stable, and functional capillary networks and that angiomas are induced only by doses above a discrete threshold level. Furthermore, within the safe range, there exists a narrower therapeutic window, whereby too low doses efficiently induce normal angiogenesis but provide no therapeutic benefit, whereas higher ones induce the growth of normal vessels of larger caliber, which are also effective in forming collateral arteries and restoring blood flow in ischemic tissue (von Degenfeld et al. 2006). This concept is reproducible also without the tedious procedure of isolating monoclonal populations. FACS purification of transduced progenitors (e.g., bone marrow- or adipose-derived mesenchymal stem cells), whose VEGF expression was suitably linked to a quantifiable cell surface marker, could rapidly generate populations producing defined and homogeneous VEGF levels (Helmrich et al. 2012). Implantation of such FACS-purified populations could avoid aberrant angiogenesis and efficiently induce only physiological and stable microvascular networks both in normal and chronically ischemic skeletal muscle (Misteli et al. 2010); (Wolff et al. 2012), as well as normal and ischemic myocardium (Melly et al. 2012, 2018).

The last key parameter to consider for therapeutically effective VEGF delivery is the duration of expression. In fact, although the development of new microvascular networks takes less than 7 days after VEGF gene delivery, the newly induced vessels are still unstable and, if VEGF signaling is lost before about 4 weeks, they can regress (Dor et al. 2002; Ozawa et al. 2004; Tafuro et al. 2009). Interestingly, the stabilization of VEGF-induced microvascular networks requires more than just pericyte recruitment, since new capillaries are already fully invested by normal pericytes 7 days after induction, but remains VEGF-dependent for a significantly longer period of time (Groppa et al. 2015). A specific population of Neuropilin1-expressing monocytes, recruited to sites of angiogenesis by Semaphorin3A secreted by activated endothelium, has been shown to play an important role in stabilizing new vessels after VEGF delivery (Groppa et al. 2015). The need to sustain VEGF delivery for about 4 weeks, but not indefinitely, is challenging for gene therapy approaches, since transient vectors (such as adenoviruses) provide expression for only about 7–10 days, whereas longer lasting vectors (such as adeno-associated and retro- or lentiviruses) do not switch off for months or even years, which raises safety concerns. The delivery of VEGF and other factors as recombinant proteins, rather than genetic information, is attractive to overcome the issues associated with heterogeneous levels in the tissue microenvironments, as a homogeneous dose distribution can be much more easily controlled (Martino et al. 2015). However, recombinant protein factors often suffer from too short half-lives in vivo. Their use in combination with biomaterials has the potential to exploit their potential and here we will specifically discuss the use of fibrin hydrogels for this purpose in regenerative medicine and therapeutic angiogenesis.

## Fibrin hydrogels for vascularization in regenerative medicine

Fibrin is derived from the soluble precursor fibrinogen during the process of blood clotting and therefore is abundantly present in normal plasma. Fibrinogen is a large fibrous glycoprotein with three pairs of polypeptide chains. The fibrinopeptides, which are in the central region, are cleaved by thrombin to convert soluble fibrinogen to insoluble fibrin monomers, which then polymerize to make fibers and branch to yield a three-dimensional network with properties of a hydrogel — the fibrin clot (Weisel 2005). Since tissue damage involves in all cases hemorrhage and blood clotting, regeneration always starts with the deposition of a fibrin-based matrix rich in growth factors (Bao et al. 2009). In fact, the three polypeptide chains of fibrin contain multiple binding sites for growth factors, cellular receptors, and integrins, which make it an ideal substrate for cell adhesion and progenitor differentiation, as well as for endothelial migration and blood vessel in-growth (Petersen et al. 2018). Fibrin also provides very attractive features for therapeutic applications, as it is injectable as a liquid and it gels in situ without cytotoxicity, is remodeled by cell-associated enzymes (e.g. metalloproteinases, plasmin), providing a provisional matrix before the deposition of tissue-specific ECM, and can also be isolated from each patient to provide an autologous material (Breen et al. 2009). Therefore, fibrin has been extensively employed for tissue engineering approaches, and it is also investigated to a more limited extent as a delivery tool for growth factors in vivo. Beyond the works discussed here by way of conceptual examples, a more extensive listing detailing the current status of fibrin use in combination with growth factors for tissue regeneration in vivo can be found in a recent review by Anitua et al. (2019).

**Fig. 1:**
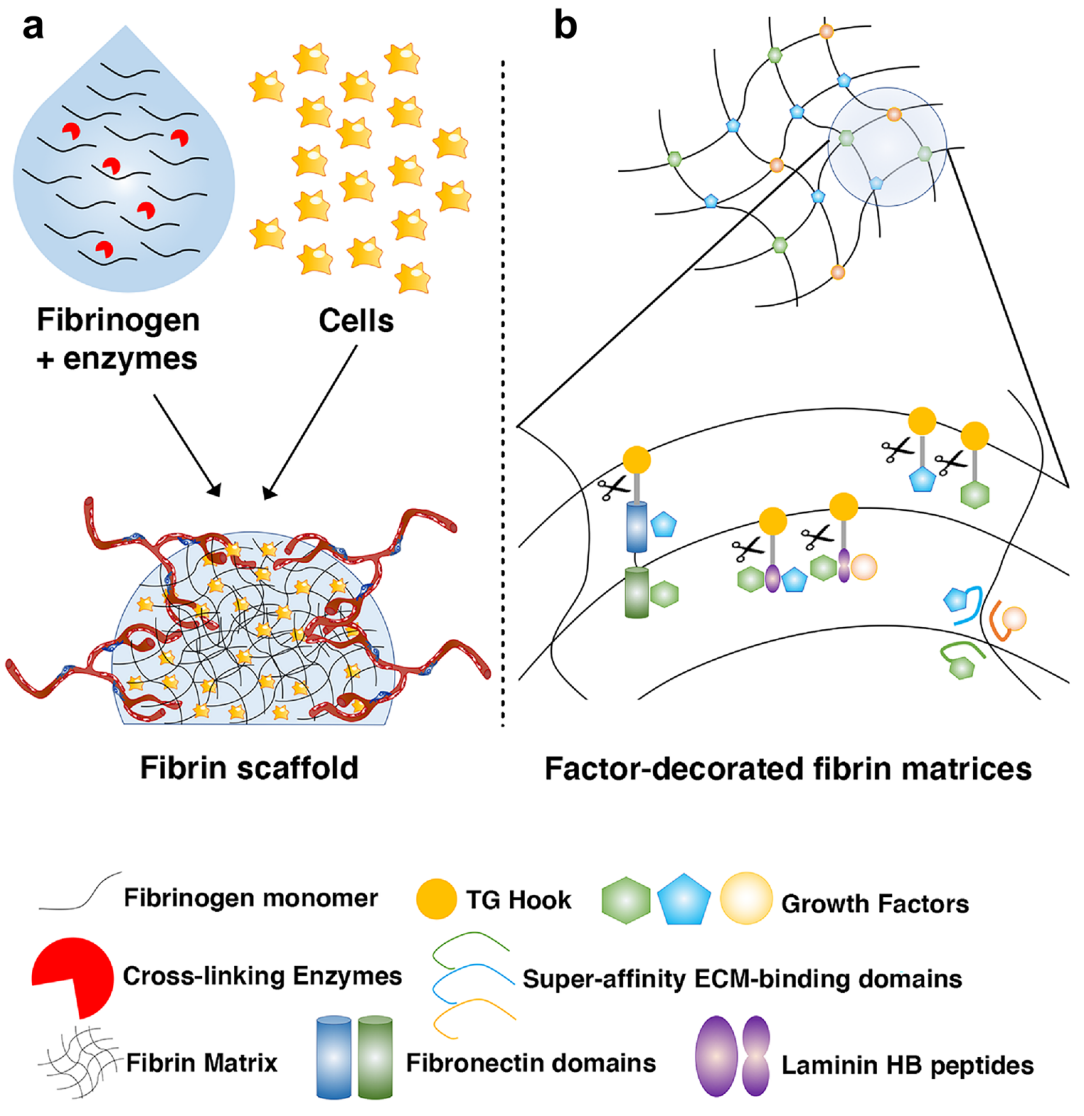
Fibrin hydrogels in regenerative medicine. **a** Fibrin hydrogels provide a transient matrix for tissue engineered grafts that mimics the provisional matrix of physiological tissue regeneration, conducive to progenitor proliferation and differentiation, as well as to rapid invasion by host-derived blood vessels. **b** Several protein engineering approaches have been developed to decorate fibrin hydrogels with recombinant morphogens and growth factors. These approaches enable the use of fibrin hydrogels as tunable platforms for controlled release of factors in vivo to guide endogenous tissue repair, as well as to provide specific morphogenic microenvironments to seeded progenitors in tissue-engineered grafts

Tissue engineering is a strategy to produce biological substitutes for tissue lost due to surgery, trauma, or degeneration and it basically relies on the combination of tissue-specific progenitors/stem cells with suitable biomaterials that provide both 3D scaffolding and a biologically appropriate environment for progenitor proliferation and differentiation. These constructs also need to rapidly attract a vascular supply after in vivo implantation, in order to survive and produce mature tissue (Gianni-Barrera et al. 2020) (Fig. 1a). The properties of fibrin described above provide several desirable features for a tissue engineering scaffold and fibrin has been used successfully to regenerate a variety of tissues, including the adipose, cardiac and skeletal muscle, bone, cartilage, skin, and liver (Ahmed et al. 2008; Anitua et al. 2019). On the other hand, limitations of fibrin hydrogels as scaffolding material derive principally from its low mechanical stiffness and short duration in vivo, with degradation by invading cells within about a week that is in some cases insufficient for proper tissue formation by seeded progenitors. For this reason, the durability of fibrin hydrogels can be significantly extended to over 4 weeks by incorporating inhibitors of fibrinolysis, such as aprotinin, which allows a significant degree of control over the degradation kinetics of the hydrogels (Sacchi et al. 2014).

Fibrin exhibits a natural affinity for some growth factors, which is functional to its role in guiding the initial steps of regeneration after injury (Martino et al. 2013). However, in order to precisely control the regenerative microenvironment, as well as to exploit fibrin as a factor-delivery platform, a variety of technologies have been developed to incorporate specific doses and combinations of growth factors within fibrin hydrogels and to control their rate and mode of release (Fig. 1b).

Taking advantage of the natural process of fibrin formation itself, growth factors have been fused to a short octapeptide sequence that is the substrate of the transglutaminase coagulation factor XIIIa (TG-hook), allowing its covalent cross-linking into fibrin hydrogels and release only by enzymatic cleavage (Zisch et al. 2001) (Ehrbar et al. 2004). By also including a TG–fused variant of the fibrinolysis inhibitor aprotinin (Lorentz et al. 2011), it was possible to independently control dose and duration of delivery in vivo of a growth factor like VEGF, developing fibrin hydrogels into a tunable factor release platform (Sacchi et al. 2014). In fact, for any given factor concentration in the hydrogel, the effective released dose per unit of time is directly proportional to the degradation rate of the matrix, while the duration of release is inversely proportional to the same parameter. Therefore, by fine-tuning TG-aprotinin concentrations, it was possible to sustain therapeutic delivery of a 500-fold dose range of VEGF in both normal and ischemic muscle over 4 weeks, as well as in a wound-healing model, with significant improvements in angiogenesis, tissue perfusion, and healing rate (Sacchi et al. 2014).

Different domains of ECM proteins have a promiscuous affinity for different growth factors. This property has been exploited to endow fibrin hydrogels with the ability to bind non-covalently, retain, and release gradually a variety of wild-type and endogenous factors, by cross-linking TG-hook-fused versions of specific domains of ECM proteins. Examples of this strategy include a multi-functional recombinant fragment of fibronectin, integrating both factor-binding and integrin-binding domains (Martino et al. 2011) and short sequences from the heparin-binding domains of the laminin α-chain (Ishihara et al. 2018).

Alternatively, engineering of any growth factor with a peptide derived from placenta-derived growth factor-2 (PlGF-2) endows them with super-affinity for a broad range of ECM proteins, including fibrin, and also enables in vivo decoration of endogenous matrix with exogenously provided therapeutic proteins (Martino et al. 2014). The increased efficacy of the modified factors avoided the need to deliver supra-physiological doses, thereby also increasing safety, while effectively promoting diabetic wound healing and bone tissue repair.

## Fibrinogen-induced fibrosis — all is not so quiet on the fibrin front

**Fig. 2:**
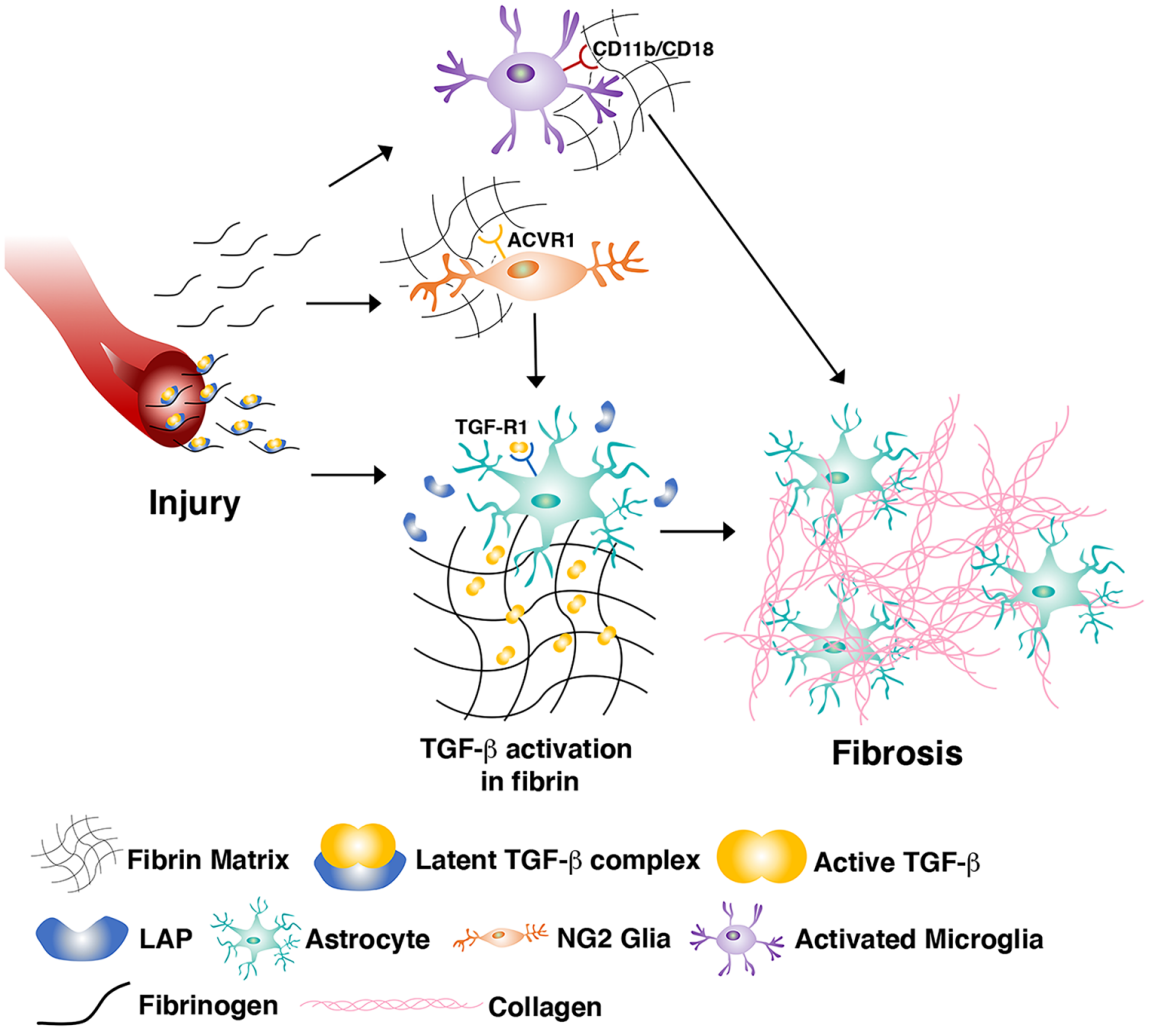
The mechanisms of fibrinogen-induced CNS fibrosis. Fibrinogen can both directly and indirectly influence the function of several cell types involved in CNS fibrosis after vascular injury. Some of the best understood interactions are summarized here: (1) activation of microglia through binding of the integrin receptor CD11b/CD18; (2) differentiation of NG2 + progenitors to an astrocyte fate through activation of the activin A receptor type I (ACVR1) and BMP signaling; and (3) activation of latent TGF-β, bound to extravasated fibrinogen, which stimulates astrocytes to initiate matrix deposition and scar formation via Smad2 signaling. LAP, latency-associated peptide; TGF-R1, TGFβ-receptor 1

Alongside all the useful features described above, it has to be mentioned that, in some cases, fibrin can be associated with undesirable outcomes, particularly the promotion of fibrosis and scar formation that actually prevent physiological tissue regeneration. This phenomenon has been especially described in the central nervous system, where scar formation starts within hours after traumatic injury. Glial scar formation is a complex process, where important roles are played by at least three major cell populations: reactive astrocytes, NG2 glia, and microglia (Adams and Gallo 2018). Fibrinogen/fibrin that leaks into the CNS after vascular injury and disruption of the blood–brain barrier has pleiotropic effects on all these cells, both directly and indirectly (Fig. 2). For example, direct fibrin effects include activation of microglia by binding the CD11b/CD18 integrin receptor, and the switch of NG2 glial progenitor differentiation from re-myelinating oligodendrocytes to reactive astrocytes, through activation of their activin A receptor type I (ACVR1) and BMP signaling (Petersen et al. 2018). Indirect actions of fibrin to promote glial scar formation have been identified in elegant work by Schachtrup and colleagues (Schachtrup et al. 2010). In fact, circulating fibrinogen is associated with latent TGF-β, which accumulates at the sites of vascular injury, is activated by interaction with astrocytes, and stimulates them to initiate scar formation via the Smad2 signaling pathway. This key role was shown by the fact that not only fibrinogen injection in the CNS was sufficient to induce scar formation in the absence of injury but also that genetic or pharmacologic depletion of fibrinogen, as well as inhibition of TGF-β signaling, could abolish scar formation after injury. More recently, fibrin deposited after CNS injury has been shown to also stimulate neural stem cell differentiation to astrocytes over neurogenesis via BMP signaling (Pous et al. 2020).

Fibrin-induced fibrosis does not appear to be a phenomenon unique to the CNS. In fact, we recently found that fibrin hydrogels promote scar formation in the myocardium, while at the same time jeopardizing the therapeutic effect of angiogenic factor delivery (Melly et al. 2020). Fibrosis was not due to the presence of growth factors, tissue damage from the injection, the mechanical stiffness of the hydrogel or its volume, nor to an immune reaction. Rather, the presence of fibrin per se was enough to start the myocardial scarring process. Interestingly, despite the fact that fibrin hydrogels were completely degraded within 5 days, the resulting scars persisted at least 4 weeks without any sign of resolution, suggesting that, once started, the process is irreversible (Melly et al. 2020). Interestingly, a recent report by the group of Serena Zacchigna shows that treatment with a monoclonal antibody blocking a specific BMP1 isoform (BMP1.3) can reduce cardiac fibrosis and scar formation in models of myocardial infarction, in part through downregulation of TGF-β signaling and myofibroblast activation (Vukicevic et al. 2022).

On the other hand, the usefulness of fibrin matrices for therapeutic factor delivery is established by solid evidence, as fibrin-induced fibrosis was not observed in a variety of other tissues and disease models (Anitua et al. 2019). For example, controlled VEGF delivery via the same fibrin-based platform in both normal and ischemic skeletal muscle, which is a contractile tissue like the myocardium or in ischemic skin wounds in mice, induced in all cases robust and functional angiogenesis without any sign of scar or fibrosis, restored blood flow and promoted healing (Sacchi et al. 2014). No skin fibrosis has ever been reported despite investigation of several fibrin-based matrices for growth factor delivery in diabetic wound healing (Whelan et al. 2014). Bladder smooth muscle has also been targeted with a bioactive fibrin-based bulking material to treat urinary incontinence, without any report of ensuing fibrosis (Vardar et al. 2019).

## Conclusions

Fibrin is the natural matrix that provides the first environment for the initiation of all forms of repair after injury. Not surprisingly, it provides attractive biological conditions for the therapeutic delivery of progenitor cells and morphogens in many fields of regenerative medicine. The specific combinations and doses of factors and duration of delivery will likely depend on the specific tissue and clinical application, as well as the possible inclusion of vascular cells in the matrix to provide more rapid vascularization and paracrine factors. For example, chronic skin wounds in diabetic patients present rather different features than large volumes of ischemic muscle in peripheral artery disease, and these are expected to require tailored therapeutic approaches. The flexible tools afforded by fibrin-based delivery platforms can be very useful to investigate the individual therapeutic needs. However, in some conditions, fibrin can also activate anti-regenerative processes, stimulating fibrosis and scar formation, with impairment of angiogenesis and stem cell differentiation. What mechanisms may tip the balance and specify one outcome or the other is still poorly understood, despite recent advances. Elucidating the molecular underpinning of fibrin-induced fibrosis will be paramount to overcome the scarring process and enable the exploitation of this attractive therapeutic platform for regenerative medicine.
